# Granulocyte-macrophage colony-stimulating factor and tumor necrosis factor-α in combination is a useful diagnostic biomarker to distinguish familial Mediterranean fever from sepsis

**DOI:** 10.1186/s13075-021-02644-2

**Published:** 2021-10-15

**Authors:** Tomohiro Koga, Kaori Furukawa, Kiyoshi Migita, Shimpei Morimoto, Toshimasa Shimizu, Shoichi Fukui, Masataka Umeda, Yushiro Endo, Remi Sumiyoshi, Shin-ya Kawashiri, Naoki Iwamoto, Kunihiro Ichinose, Mami Tamai, Tomoki Origuchi, Takahiro Maeda, Akihiro Yachie, Atsushi Kawakami

**Affiliations:** 1 Department of Immunology and Rheumatology, Division of Advanced Preventive Medical Sciences, Nagasaki University Graduate School of Biomedical Sciences, Nagasaki, Japan; 2 Center for Bioinformatics and Molecular Medicine, Nagasaki University Graduate School of Biomedical Sciences, 1-12-4 Sakamoto, Nagasaki, 852-8523 Japan; 3 Department of Rheumatology, Fukushima Medical University School of Medicine, Fukushima, Japan; 4 Nagasaki University Hospital, Clinical Research Center, Nagasaki, Japan; 5 Department of General Medicine, Nagasaki University Hospital, Nagasaki University Graduate School of Biomedical Sciences, Nagasaki, Japan; 6 Department of Community Medicine, Nagasaki University Graduate School of Biomedical Sciences, Nagasaki, Japan; 7 Division of Medical Safety, Kanazawa University Hospital, Kanazawa, Japan

**Keywords:** Familial Mediterranean fever, sepsis, TNF-α, GM-CSF, Cytokine profile

## Abstract

**Objective:**

To identify potential biomarkers to distinguish familial Mediterranean fever (FMF) from sepsis.

**Method:**

We recruited 28 patients diagnosed with typical FMF (according to the Tel Hashomer criteria), 22 patients with sepsis, and 118 age-matched controls. Serum levels of 40 cytokines were analyzed using multi-suspension cytokine array. We performed a cluster analysis of each cytokine in the FMF and sepsis groups in order to identify specific molecular networks. Multivariate classification (random forest analysis) and logistic regression analysis were used to rank the cytokines by importance and determine specific biomarkers for distinguishing FMF from sepsis.

**Results:**

Fifteen of the 40 cytokines were found to be suitable for further analysis. Levels of serum granulocyte-macrophage colony-stimulating factor (GM-CSF), fibroblast growth factor 2, vascular endothelial growth factor, macrophage inflammatory protein-1b, and interleukin-17 were significantly elevated, whereas tumor necrosis factor-α (TNF-α) was significantly lower in patients with FMF compared with those with sepsis. Cytokine clustering patterns differed between the two groups. Multivariate classification followed by logistic regression analysis revealed that measurement of both GM-CSF and TNF-α could distinguish FMF from sepsis with high accuracy (cut-off values for GM-CSF = 8.3 pg/mL; TNF-α = 16**.**3 pg/mL; sensitivity, 92.9%; specificity, 94.4%; accuracy, 93.4%).

**Conclusion:**

Determination of GM-CSF and TNF-α levels in combination may represent a biomarker for the differential diagnosis of FMF from sepsis, based on measurement of multiple cytokines.

**Supplementary Information:**

The online version contains supplementary material available at 10.1186/s13075-021-02644-2.

## Introduction

Familial Mediterranean fever (FMF) is an inherited autoinflammatory disease clinically characterized by periodic fevers with serositis [1, 2]. The gene responsible for the disease is the *MEFV* gene, and the pathogenesis of the disease is related to hyperinflammation of inflammasomes due to the altered function of the pyrin encoded by the *MEFV* gene [3–5]. Although there are no specific serum biomarkers for the diagnosis of FMF, previous reports have shown that serum interleukin (IL)-1β, soluble IL-2 receptor, IL-6, tumor necrosis factor-α (TNF-α), IL-10, IL-12, IL-17A, and IL-18 are important for the pathogenesis of FMF [6–12].

Autoinflammatory diseases, including FMF, are important to differentiate from unknown fever. Distinguishing FMF from bacterial infections, including sepsis, is difficult in some cases in daily practice. Delayed diagnosis of sepsis often leads to severe conditions, including disseminated intravascular coagulation, which can be life-threatening due to multiple organ failure. In addition, although genetic testing is important for FMF, there are FMF cases that do not have the *MEFV* gene variant.

It has been reported that serum cytokines such as IL-1β, IL-6, IL-8, IL-10, IL-12, IL-17A, IL-18, interferon gamma (IFN-γ), and TNF-α are elevated in sepsis [13–16], but some of these cytokines are also elevated in FMF patients. It is not clear whether existing biomarkers are useful in differentiating between these diseases, and few studies have compared cytokines in FMF and sepsis patients.

To solve this problem, we have attempted to identify useful biomarkers for differentiation by comprehensively analyzing the serum cytokine profiles of both diseases and comparing them in detail using machine learning direction including decision tree analysis.

## Methods

### Patients and controls

This study was registered with the University Hospital Medical Information Network Clinical Trials Registry (http://www.umin.ac.jp/ctr) as UMIN000030922. We prospectively recruited consecutive patients with FMF who were treated at Nagasaki University, Shinshu University, and Kanazawa University between April 2014 and March 2019. Diagnosis of FMF was based on the Tel Hashomer criteria [17, 18]. We also recruited patients with sepsis who were admitted to the Rheumatology Department of Nagasaki University Hospital between April 2016 and October 2018 and required differential diagnoses from fever of unknown origin (FUO). These patients did not have any other underlying rheumatological conditions. Sepsis was defined according to the Sepsis-3 Criteria—increase in Sequential Organ Failure Assessment score of ≥ 2 at day 1 and suspicion of infection [19]. All participants underwent clinical assessment and provided blood samples for analysis at the time of admission. The control group was recruited from staff at Nagasaki University and residents of the town of Saza in Nagasaki Prefecture, as previously described [20].

All patients provided written informed consent for participation, and the study and all its protocols were approved by the Institutional Review Board of Nagasaki University and related centers (Approval No. 18011512-4). Studies involving residents of Saza were approved by the Nagasaki University Ethics Committee for Human Use (Approval No. 14051404). Written informed consent was obtained from residents of Saza, who underwent specific health checkups.

### *MEFV* gene sequencing

*MEFV* genetic analysis was performed on all patients in this study. Promega Wizard® Genomic DNA Purification Kit (Promega, Madison, WI, USA) was used to extract genomic DNA from blood samples. We subsequently performed polymerase chain reaction (PCR) using the forward and reverse primers for each exon of the *MEFV* gene, as previously described [21]. We purified PCR products with the reagent ExoSAP-IT™ (GE Healthcare Japan, Tokyo, Japan) and sequenced directly, using specific primers and BigDye Terminator v1.1 (Applied Biosystems, Tokyo, Japan).

### Multiplex cytokine and chemokine bead assays

Serum samples were centrifuged at 3000 × g for 5 min, and the supernatants collected and stored at − 80 °C for a maximum of 90 days prior to analysis. A blinded multiplex cytokine bead assay was performed in parallel using the Bio-plex MAGPIX™ Human Cytokine Assay (Bio-Rad, Hercules, CA, USA) and MILLIPLEX® MAP Human Cytokine/Chemokine Magnetic Bead Panel 1-Premixed 38 Plex (Millipore, Billerica, MA, USA) kits, according to the manufacturers’ instructions. Cytokines that were frequently found to be at nondetectable levels were excluded from analysis. The multiplex cytokine bead assays in this study was done in two times, and we calculated the coefficient of variations (CVs) for each cytokine using the quality controls samples in the first and second times.

### Statistical analysis

Baseline demographic characteristics and cytokine/chemokine levels of the study population were compared using the Kruskal–Wallis test, followed by Dunn’s multiple comparisons test. Correlations between pairs of serum markers were calculated using Spearman’s rank correlation test. To rank the cytokine levels, we performed the multivariate classification algorithm of Random Forest analysis (RFA) using the R software package RandomForest (http://cran.r-project.org/web/packages/randomForest/) version 4.6.12, as previously described [20]. We subsequently selected a classifier, consisting of a combination of cytokine markers that yielded the best classification performance to predict FMF, using multivariable logistic regression analysis. We then calculated the sensitivity, specificity, accuracy, receiver operator characteristic (ROC) curve, area under the curve (AUC), and Akaike’s information criterion (AIC). Statistical analyses were performed using R software (version 4.1.0) and JMP pro (version 15.0) (SAS Institute, Cary, NC, USA). All reported *p* values are two-sided, and a *p* value of < 0.05 was considered statistically significant. The family-wise error rate in multiple hypotheses testing was considered by shrinking the size of a test by dividing the original size of a test by the size of the family of the tests; the respective applications are stated in the Results or footnotes in tables.

## Results

### Study population

The study population comprised 28 patients with FMF, 22 with sepsis, and 118 age- and sex-matched healthy controls. Table 1 presents the demographic and clinical characteristics of the patients with FMF and sepsis. The median ages at diagnosis were 40 years and 68 years in the FMF and the sepsis groups, respectively. All patients with FMF had *MEFV* genetic testing; the percentage of patients with the M694I variant in exon 10 was 39%.

**Table 1:** The demographic, clinical, and laboratory characteristics of the FMF patients, and the sepsis patients

Characteristic	FMF patients (***n*** = 28)	Sepsis patients (***n*** = 22)	Healthy controls (***n*** = 118)
Age at onset, years	24 (9–48)		
Age at diagnosis, years	40 (29–50)	59 (44–71)	56 (47–65)
Female, *n* (%)	19 (68)	12 (54)	11 (58)
Serum CRP, mg/L	63 (44–99)*	116 (99–148)**	
SOFA score		3 (2.8–4.3)	
DIC, *n* (%)	0 (0)	3 (18)	
MAS, *n* (%)	0 (0)	0 (0)	
M694I mutation, *n* (%)	11 (39)		
Typical FMF, *n* (%)	21 (75)		

*CRP* C-reactive protein; *SOFA* sequential organ failure assessment; *DIC* disseminated intravascular coagulation; *MAS* macrophage activation syndrome. *n = 6, **n = 10

### Cytokine profiles of patients with FMF, sepsis, and healthy controls

After exclusion of cytokines that were frequently nondetectable, we were able to analyze 15 cytokines: fibroblast growth factor 2 (-2), basic granulocyte colony-stimulating factor (G-CSF), granulocyte-macrophage colony-stimulating factor (GM-CSF), CXCL1 (growth-regulated protein alpha precursor [GRO]), IFN-γ, IL-17A, IL-18, IL-6, IL-8, CXCL10 (interferon gamma-inducible protein 10 [IP-10]), CCL2 (monocyte chemoattractant protein-1 [MCP-1]/MCAF), CCL3 (macrophage inflammatory protein-1α [MIP-1α]), CCL4 (macrophage inflammatory protein-1β [MIP-1β]), TNF-α, and vascular endothelial growth factor (VEGF).

**Fig. 1:**
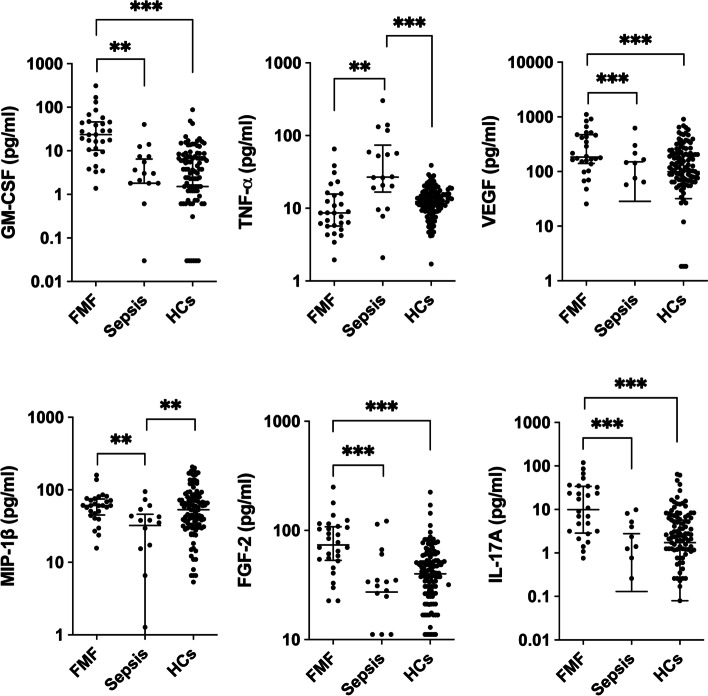
A multiplex cytokine bead assay of GM-CSF, TNF-α, VEGF, MIP-1β, FGF-2, and IL-17A in the serum of patients with FMF and sepsis. Data are presented as individual plots (median, interquartile range). ***p < 0.001, **p < 0.01. GM-CSF, granulocyte-macrophage colony-stimulating factor; TNF-α, tumor necrosis factor-α; VEGF, vascular endothelial growth factor; MIP-1β, macrophage inflammatory protein-1b; FGF-2, fibroblast growth factor 2; IL-17A, interleukin-17; FMF, familial Mediterranean fever

Serum levels of six cytokines were significantly elevated in patients with FMF compared with those with sepsis (median FGF-2 73.8 pg/mL vs. 25.7, *p* < 0.0001; median GM-CSF 23.5 pg/mL vs. 1.8 pg/mL, *p* < 0.0001; median IL-17A: 9.9 pg/mL vs. 0.13 pg/mL, *p* < 0.0001; median MIP-1β 60.8 pg/mL vs. 32.4 pg/mL, *p* = 0.0016; median TNF-α 8.6 pg/mL vs. 26.9 pg/mL, *p* < 0.0010; and median VEGF 184 pg/mL vs. 28.6 pg/mL, *p* = 0.0002) (Fig. 1). Among the six cytokines that differed significantly between the sepsis and FMF groups, GM-CSF, VEGF, FGF-2, and IL-17A were significantly increased in the FMF group compared with the control group, and TNF-α and MIP-1β were significantly higher in the sepsis group than in healthy subjects (Fig. 1). Eight cytokines (FGF-2, G-CSF, GM-CSF, IFN-γ, IL-17A, IL-6, CXCL10, and VEGF) were significantly increased in the FMF group compared with the control group (all *p* values are *p* < 0.0001, Table 2). On the other hand, the levels of five cytokines (G-CSF, IL-6, CXCL10, MCP-1, and TNF-α) were significantly higher in the septic group than in the control group (*p* < 0.0001, *p* < 0.0001, *p* < 0.0001, *p* = 0.0005, *p* = 0.0002, respectively, Table 2). The inter-assay CVs in this study were within 10% for all cytokines, which was in close agreement with the CVs provided by the manufacturer. The CVs of GM-CSF and TNF-α, two particularly important cytokines, were 0.38% and 4.0%, respectively.

**Table 2:** Cytokine profile of FMF patients, sepsis patients, and healthy controls

	FMF			Sepsis			HCs			***p*** value		
	Median	25th	75th	Median	25th	75th	Median	25th	75th	FMF vs. sepsis	Sepsis vs. HCs	FMF vs. HCs
**FGF-2**	73.8	53.1	108	25.7	0	34.1	37.7	21.2	52.2	**< 0.001**	0.016	< 0.001
**G-CSF**	36.9	22.5	75.0	41.2	10.4	149	8.2	0	23.0	0.99	< 0.0001	< 0.0001
**GM-CSF**	23.5	10.8	46.6	1.8	0	3.2	1.5	0	6.7	**< 0.001**	0.41	< 0.0001
**GRO**	1193	679	1521	967	516	1453	969	750	1204	0.63	0.99	0.22
**IFN-g**	15.4	7.2	52.6	4.1	0.8	20.8	4.6	1.3	9.8	0.0313	0.98	< 0.0001
**IL-17A**	9.9	2.9	33.9	0.13	0	2.8	1.93	0.1	5.8	**< 0.001**	0.11	< 0.0001
**IL-6**	23.7	5.5	49.0	34.7	0.7	61.1	0	0	0	0.71	< 0.0001	< 0.0001
**IL-8**	33.1	16.0	44.5	30.8	13.3	55.4	63.03	23.5	108	0.94	0.031	0.048
**IP-10**	333	185	950	2083	542	4212	271.68	221	352	0.049	< 0.0001	< 0.0001
**MCP-1**	453	318	752	1263	805	1708	656	526	837	0.015	0.0005	0.023
**MIP-1a**	10.9	0.2	16.4	6.8	0	24.5	12.4	0	27.8	0.89	0.53	0.28
**MIP-1b**	60.8	45.1	74.8	32.4	1.0	46.0	53.2	31.1	79.4	**0.0016**	0.0077	0.69
**TNF-α**	8.6	5.7	15.7	26.9	16.7	73.9	12.4	9.3	16.6	**0.0010**	0.0002	0.055
**VEGF**	184	141	472	28.6	0	151	88.5	26.6	200	**0.0002**	0.083	< 0.0001
**IL-18**	94.2	50.6	772	105	75.1	382	112	65.2	181	0.84	0.86	0.71

Values are the median (interquartile range) pg/ml. *p* values were established using Kruskal-Wallis test followed by a Dunn’s multiple comparisons test. *FGF* fibroblast growth factor; *GM-CSF* granulocyte-macrophage colony-stimulating factor; *GRO* growth-regulated protein alpha precursor; *G-CSF* granulocyte colony-stimulating factor; *IL* interleukin; *MCP-1* monocyte chemoattractant protein-1; *TNF-α* tumor necrosis factor-alpha; *VEGF* vascular endothelial growth factor. Bonferroni’s correction for multiple-cytokine testing was applied and *p* < 0.0033 was considered significant

### Comparison of cytokine networks between patients with FMF and patients with sepsis

To compare cytokine networks between patients with FMF and those with sepsis, we further examined the correlations between serum levels of individual cytokines in patients with FMF and in patients with sepsis. We found significant correlations between FGF-2 and GM-CSF (*ρ* = 0.755, *p* < 0.0001), FGF-2 and VEGF (*ρ* = 0.500, *p* < 0.0001), FGF-2 and MIP-1β (*ρ* = 0.457, *p* < 0.0001), MIP-1β and GM-CSF (*ρ* = 0.412, *p* < 0.0001), TNF-α and IP-10 (*ρ* = 0.406, *p* < 0.0001), and TNF-α and MIP-1β (*ρ* = 0.240, *p* = 0.0020) in the FMF group.

**Fig. 2:**
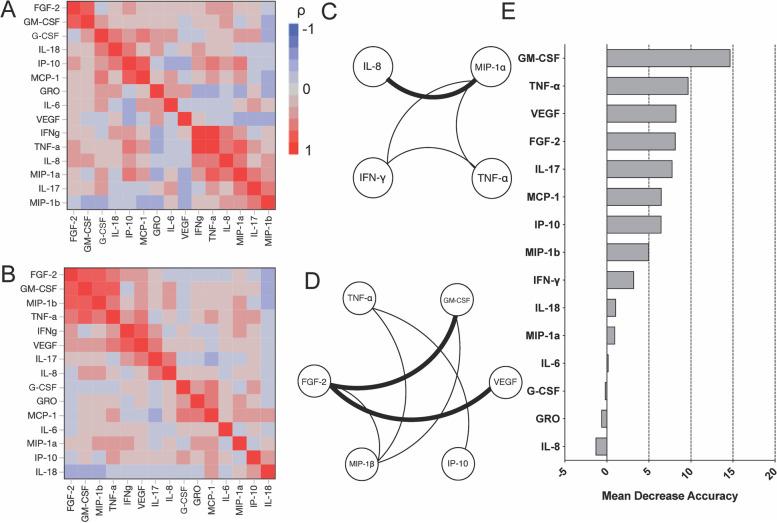
Cytokine networks in the patients with FMF and sepsis. Hierarchical clustering with a Spearman’s rank correlation heatmap of serum cytokine levels among patients with **A** sepsis and **B** FMF. Serum circular network layouts in **C** sepsis and **D** FMF. Significant correlations (*p* < 0.0033) were represented by connecting edges to underscore strong positive (*ρ* > 0.50; thick black line), moderate positive (0.3 < *ρ* < 0.5; thin black line). **E** Cytokines ranked by their relative importance for discriminating FMF from sepsis. The horizontal axis represents the average decrease in classification accuracy. FMF, familial Mediterranean fever

In the sepsis group, significant correlations were found between IL-8 and MIP-1α (*ρ* = 0.549, *p* < 0.0001), TNF-α and MIP-1α (*ρ* = 0.374, *p* < 0.0001), MIP-1α and IFN-γ (*ρ* = 0.367, *p* < 0.0001), and TNF-α and IFN-γ (*ρ* = 0.234, *p* = 0.0021). Hierarchical clustering with heatmaps based on the Spearman’s rank correlation test is shown in Fig. 2A (for the FMF group) and Fig. 2B (for the sepsis group). Considering the correlation coefficients, we constructed a circular network layout as shown in Figure 2C, D. Compared to the sepsis group (Fig. 2C), the FMF group (Fig. 2D) showed more complex crosstalk between the molecular species in the network with strong correlation edges.

These results suggest that cytokine networks in patients with FMF differ from those in patients with sepsis. Thus, in the FMF group, FGF-2, GM-CSF, MIP-1β, and TNF-α form interrelated networks, whereas in the sepsis group, IFN-γ, TNF-α, IL-8, and MIP-1α form interrelated networks.

### Identification of combinational biomarkers for the differential diagnosis of FMF from sepsis by RFA and logistic regression analysis

**Fig. 3:**
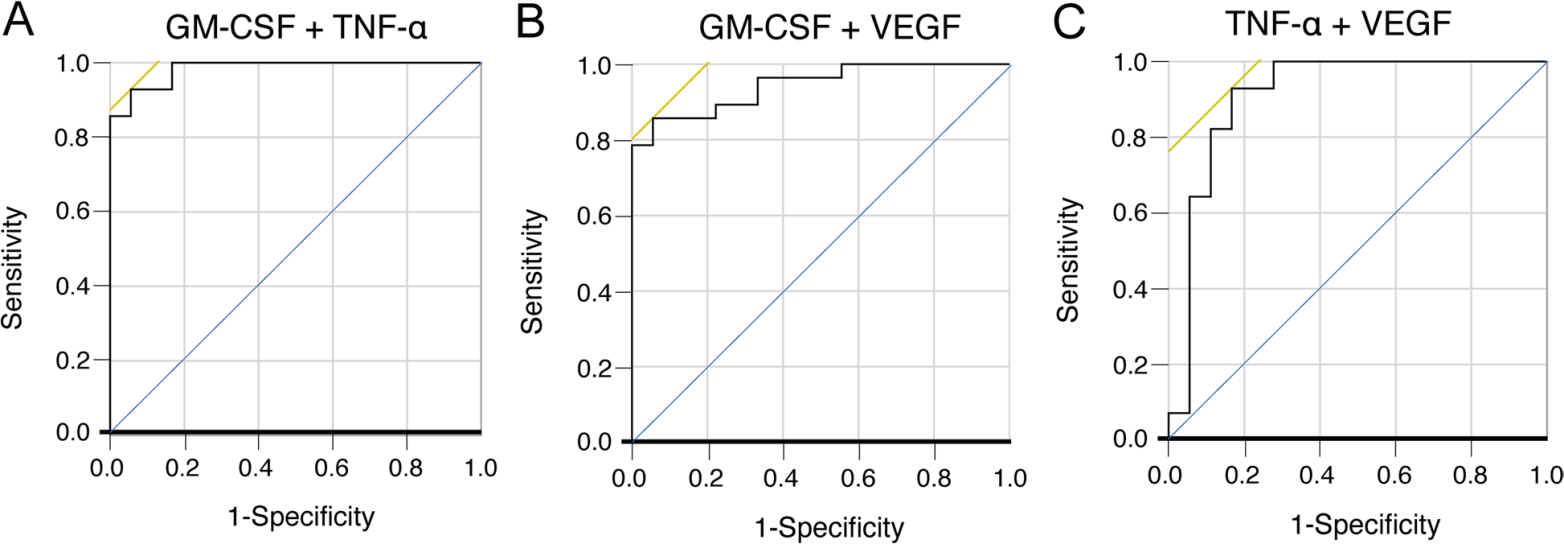
ROC curve analysis for the prediction of FMF by a specific set of cytokines. **A** The combined measurement of GM-CSF and TNF-α. **B** The combined measurement of GM-CSF and VEGF. **C** The combined measurement of TNF-α and VEGF. GM-CSF, granulocyte-macrophage colony-stimulating factor; TNF-α, tumor necrosis factor-α; VEGF, vascular endothelial growth factor

The results of ranking of cytokines by importance, according to RFA, are illustrated in Fig. 1C. GM-CSF and TNF-α were extracted as the most important cytokines for distinguishing FMF from sepsis (mean decrease accuracy 14.7 and 9.7, respectively). The results of multivariable logistic regression analysis and ROC curves for sensitivity, specificity, accuracy, AUC, and AIC are shown in Table 3 and Fig. 3A–C. The best combination of cytokines to distinguish FMF from sepsis was found to be GM-CSF and TNF-α, with high accuracy observed (sensitivity 93%, specificity 94%, and accuracy 93%; Table 3). We selected these variables for a logistic regression analysis and identified independent prognostic factors of FMF, as follows: GM-CSF (1.0-unit increase, odds ratio [OR] = 1.56, 95% confidence interval [CI] 1.05–2.32, *p* < 0.0001) and TNF-α (1.0-unit increase, OR = 0.77, 95%CI 0.59–1.01, *p* = 0.0003). After adjusting for the effect of age on cytokines by adding age as an explanatory factor, the results were similar for sensitivity, specificity, and positive detection rates for each cytokine combination (Supplementary Table 1).

**Table 3:** ROC curve in each subset determined by univariate/multivariable logistic regression analysis

Variables (FMF vs. sepsis)	Sensitivity	Specificity	Accuracy	AUC	AIC	Cutoff value (pg/mL or predicted probability*)
GM-CSF	0.86	0.94	0.89	0.95	30.3	8.3
TNF-α	0.82	0.78	0.80	0.82	62.7	16.3
VEGF	0.90	0.75	0.82	0.83	54.3	67.1
GM-CSF + TNF-α	0.93	0.94	0.93	0.98	19.3	0.75*
GM-CSF + VEGF	0.86	0.94	0.89	0.94	32.3	0.61*
TNF-α + VEGF	0.93	0.83	0.89	0.91	42.4	0.55*

Bold indicates the minimum number of cytokines among the subsets. *AIC* Akaike’s information criterion; *AUC* area under the curve; *GM-CSF* granulocyte-macrophage colony-stimulating factor; *TNF-α* tumor necrosis factor-alpha; *VEGF* vascular endothelial growth factor

## Discussion

We have previously shown that the combination of serum IL-18 and FGF-2 is a useful biomarker to discriminate between sepsis and adult-onset Still’s disease (AOSD) [20]. By adapting a similar approach to FMF patients, we extracted GM-CSF and TNF-α as biomarkers that discriminate between FMF and sepsis. Although both FMF and AOSD are classified as autoinflammatory diseases and the elevated cytokines and pathophysiology are partly similar [22, 23], it was surprising that the results of extracted cytokines were different between AOSD and FMF patients. When comparing FMF and AOSD, elevated IL-18 was characteristic of AOSD [23–26]; this difference in IL-18 levels may have been reflected in the results of the different cytokine combinations in the two diseases.

In the present study, serum GM-CSF was significantly lower in sepsis patients compared with healthy controls. Consistent with the results of this study, plasma GM-CSF in sepsis is significantly lower in the nonsurvivor group than in survivors and healthy controls [27]. In septic patients, GM-CSF has also been shown to act protectively by restoring or improving human monocyte function, and administration of GM-CSF in mice has been shown to prevent abdominal sepsis [28–30]. Although the detailed mechanism by which GM-CSF production is reduced by bacterial infection has not been established, it is interesting to note that these changes contrast with the levels in FMF patient sera.

The cytokine profile of FMF is based on aberrant activity of inflammasomes. Because of the production of IL-1 and IL-18 by macrophages and neutrophils, T cells and vascular endothelial cells are activated, resulting in a diverse cytokine pattern. In this study, GM-CSF levels were significantly higher in FMF patients than in sepsis patients and healthy subjects. GM-CSF is secreted by various cells, including macrophages, T cells, and vascular endothelial cells, which may be different from the pathogenesis of sepsis. It has been recently reported that GM-CSF activates inflammasomes in THP-1 cells and human neutrophils via JAK2 signaling, which may reflect the pathogenesis of FMF [31]. In this study, in addition to serum GM-CSF, MIP-1β was significantly higher in FMF patients than in septic patients. This result may reflect the activation of macrophages in FMF, which may reflect inflammation during the attack period. Considering that FMF promotes activation of inflammasomes due to dysfunctions of pylin protein, it is suggested that macrophages, the cells most associated with inflammasomes, are activated during the attack phase of FMF. However, although a previous report has shown that GM-CSF is significantly higher in the attack phase than in remission phases of FMF [6], there is no difference in MIP-1β between the previous report and the results of this study [6]. In the present study, MIP-1β was significantly lower in patients with sepsis than in healthy subjects, but the mechanism of this is not clear and should be investigated in the future.

There have been several studies on sepsis in which serum cytokines have been analyzed. It was reported that the cytokines IL-1β, IL-4, IL-6, IL-8, MCP-1, and G-CSF had good accuracy for predicting early mortality (< 48 h), and IL-8 and MCP-1 had the best accuracy for predicting mortality at 28 days [13]. In addition, serum cytokine levels of IL-1β, IL-6, IL-8, IL-10, IL-12, IL-17A, IL-18, IFN-γ, TNF-α, and calprotectin were found to be elevated in sepsis [14, 16, 32]. The levels of cytokines IL-1β, IL-6, IL-8, MCP-1, and IL-10, and of plasminogen activator inhibitor 1 (PAI-1), were reported to be increased over the acute phase and that IL-6, IL-8, MCP-1, and IL-10 formed a cytokine network in the acute phase of sepsis [15]. The results of the present study were consistent with previous reports, in that IL-6, IL-8, MCP-1, G-CSF, IFN-γ, and TNF-α were higher than in healthy subjects, but IL-18 and IL-17A were not significantly different between healthy subjects and sepsis patients.

In the present study, serum TNF-α levels in patients with FMF during attacks were not significantly different than those in healthy subjects. Comparing these results with previous cytokine analyses using patient sera during the attack phase of FMF, there are reports that there is no difference in TNF-α levels in FMF patients compared to healthy subjects [6, 7], while there are also reports that serum TNF-α is elevated in FMF [9]. Since TNF inhibitors are effective in FMF patients, it is quite possible that TNF-α reflects the pathogenesis of FMF, but in this study, we could not detect any significant difference in serum. As for the elevation of TNF-α in septic patients, the results were elevated in this study, as well as in previous reports.

Serum GM-CSF levels have been shown to be lower in the elderly [33]. In the present study, there were more elderly patients with sepsis than those with FMF, so the possibility of an age effect cannot be ruled out. On the other hand, other cytokines that were significantly different between the two groups (TNF-α, VEGF, FGF-2, MIP-1β, and IL-17A) were not affected by age [33].

The strength of this study is that it is, to the best of our knowledge, the first to identify clinically important serum biomarkers useful for differentiating sepsis from FMF, using a Japanese cohort in which all patients were genetically tested. However, it should be recognized that there are several limitations of this study. Because of its cross-sectional design, serial serum samples were not used. A validation cohort for the random forest analysis was not designed in this study. The cytokine combinations identified in this study using machine learning will need to be validated in another cohort in the future. Although the biomarker may be useful in differentiating FMF from sepsis, it may not reflect therapeutic effect or disease activity. Furthermore, since the present study included patients who were hospitalized for evaluation of FUO and subsequently diagnosed with sepsis, the cytokine profile of our septic patients may differ from that in the normal population. Therefore, future studies with a larger number of septic patients should be conducted. Finally, although the efficacy of IL-1 inhibitors in patients with severe FMF has been shown [34], serum IL-1β levels in the assay we used were not high enough to detect significant differences in FMF or septic patients.

## Conclusions

In conclusion, this study showed that GM-CSF and TNF-α can be useful biomarkers for the differential diagnosis of FMF and sepsis when measured in combination. The cytokines that showed contrasting changes in sepsis and FMF will not only provide important insights into the pathogenesis of both diseases and the creation of therapeutic agents, but will also improve the ability to diagnose FMF in daily practice.

## Supplementary Information

**Additional file 1: **Supplementary **Table S1** Age adjusted ROC curve in each subset determined by multivariable logistic regression analysis

## Data Availability

The datasets used and/or analyzed during the present study are available from the corresponding author on reasonable request.

## Abbreviations

AIC: Akaike’s information criterion
AOSD: Adult-onset Still’s disease
AUC: Area under the curve
FGF-2: Fibroblast growth factor 2
FMF: Familial Mediterranean fever
FUO: Fever of unknown origin
G-CSF: Granulocyte colony-stimulating factor
GM-CSF: Granulocyte-macrophage colony-stimulating factor
IFN-γ: Interferon gamma
IL: Interleukin
MIP: Macrophage inflammatory protein
PCR: Polymerase chain reaction
RFA: Random Forest analysis
ROC: Receiver operator characteristic
TNF-α: Tumor necrosis factor-α
VEGF: Vascular endothelial growth factor

## References

[1] Ben-Chetrit E, Levy M (1998). Familial Mediterranean fever. Lancet.

[2] Livneh A, Langevitz P, Zemer D, Padeh S, Migdal A, Sohar E, Pras M (1996). The changing face of familial Mediterranean fever. Semin Arthritis Rheum.

[3] Bernot A, Clepet C, Dasilva C, Devaud C, Petit J-L, Caloustian C, Cruaud C, Samson D, Pulcini F, The French FMFC (1997). A candidate gene for familial Mediterranean fever. Nature Genetics.

[4] The International FMFC (1997). Ancient missense mutations in a new member of the RoRet gene family are likely to cause familial Mediterranean fever. Cell.

[5] Papin S, Duquesnoy P, Cazeneuve C, Pantel J, Coppey-Moisan M, Dargemont C, Amselem S (2000). Alternative splicing at the MEFV locus involved in familial Mediterranean fever regulates translocation of the marenostrin/pyrin protein to the nucleus. Hum Mol Genet.

[6] Koga T, Migita K, Sato S, Umeda M, Nonaka F, Kawashiri SY, Iwamoto N, Ichinose K, Tamai M, Nakamura H (2016). Multiple serum cytokine profiling to identify combinational diagnostic biomarkers in attacks of familial mediterranean fever. Medicine.

[7] Manukyan GP, Ghazaryan KA, Ktsoyan Zh A, Tatyan MV, Khachatryan ZA, Hakobyan GS, Mkrtchyan VA, Kelly D, Coutts A, Aminov RI (2008). Cytokine profile of Armenian patients with Familial Mediterranean fever. Clinical biochemistry.

[8] Bagci S, Toy B, Tuzun A, Ates Y, Aslan M, Inal A, Gulsen M, Karaeren N, Dagalp K (2004). Continuity of cytokine activation in patients with familial Mediterranean fever. Clinical rheumatology.

[9] Baykal Y, Saglam K, Yilmaz MI, Taslipinar A, Akinci SB, Inal A (2003). Serum sIL-2r, IL-6, IL-10 and TNF-alpha level in familial Mediterranean fever patients. Clinical rheumatology.

[10] Erken E, Ozer HT, Gunesacar R (2006). Plasma interleukin-10 and interleukin-12 levels in patients with familial Mediterranean fever. Rheumatol Int.

[11] Haznedaroglu S, Ozturk MA, Sancak B, Goker B, Onat AM, Bukan N, Ertenli I, Kiraz S, Calguneri M (2005). Serum interleukin 17 and interleukin 18 levels in familial Mediterranean fever. Clin Exp Rheumatol.

[12] Korkmaz C, Cansu DU, Cansu GB (2020). Familial Mediterranean fever: the molecular pathways from stress exposure to attacks. Rheumatology (Oxford).

[13] Bozza FA, Salluh JI, Japiassu AM, Soares M, Assis EF, Gomes RN, Bozza MT, Castro-Faria-Neto HC, Bozza PT (2007). Cytokine profiles as markers of disease severity in sepsis: a multiplex analysis. Crit Care.

[14] Rau M, Schiller M, Krienke S, Heyder P, Lorenz H, Blank N (2010). Clinical manifestations but not cytokine profiles differentiate adult-onset Still’s disease and sepsis. J Rheumatol.

[15] Matsumoto H, Ogura H, Shimizu K, Ikeda M, Hirose T, Matsuura H, Kang S, Takahashi K, Tanaka T, Shimazu T (2018). The clinical importance of a cytokine network in the acute phase of sepsis. Sci Rep.

[16] Morrow KN, Coopersmith CM, Ford ML (2019). IL-17, IL-27, and IL-33: a novel axis linked to immunological dysfunction during sepsis. Front Immunol.

[17] Livneh A, Langevitz P, Zemer D, Zaks N, Kees S, Lidar T, Migdal A, Padeh S, Pras M (1997). Criteria for the diagnosis of familial Mediterranean fever. Arthritis Rheum.

[18] Berkun Y, Eisenstein EM (2014). Diagnostic criteria of familial Mediterranean fever. Autoimmunity reviews.

[19] Singer M, Deutschman CS, Seymour CW, Shankar-Hari M, Annane D, Bauer M, Bellomo R, Bernard GR, Chiche JD, Coopersmith CM (2016). The Third International Consensus Definitions for Sepsis and Septic Shock (Sepsis-3). JAMA.

[20] Koga T, Sumiyoshi R, Furukawa K, Sato S, Migita K, Shimizu T, Umeda M, Endo Y, Fukui S, Kawashiri SY (2020). Interleukin-18 and fibroblast growth factor 2 in combination is a useful diagnostic biomarker to distinguish adult-onset Still’s disease from sepsis. Arthritis Res Ther.

[21] Migita K, Agematsu K, Yazaki M, Nonaka F, Nakamura A, Toma T, Kishida D, Uehara R, Nakamura Y, Jiuchi Y (2014). Familial Mediterranean fever: genotype-phenotype correlations in Japanese patients. Medicine (Baltimore).

[22] Priori R, Colafrancesco S, Alessandri C, Minniti A, Perricone C, Iaiani G, Palazzo D, Valesini G (2014). Interleukin 18: a biomarker for differential diagnosis between adult-onset Still’s disease and sepsis. J Rheumatol.

[23] Girard C, Rech J, Brown M, Allali D, Roux-Lombard P, Spertini F, Schiffrin EJ, Schett G, Manger B, Bas S (2016). Elevated serum levels of free interleukin-18 in adult-onset Still’s disease. Rheumatology (Oxford).

[24] Feist E, Mitrovic S, Fautrel B (2018). Mechanisms, biomarkers and targets for adult-onset Still’s disease. Nature reviews Rheumatology.

[25] Chen DY, Lan JL, Lin FJ, Hsieh TY (2004). Proinflammatory cytokine profiles in sera and pathological tissues of patients with active untreated adult onset Still’s disease. J Rheumatol.

[26] Choi JH, Suh CH, Lee YM, Suh YJ, Lee SK, Kim SS, Nahm DH, Park HS (2003). Serum cytokine profiles in patients with adult onset Still’s disease. J Rheumatol.

[27] Perry SE, Mostafa SM, Wenstone R, McLaughlin PJ (2002). Low plasma granulocyte-macrophage colony stimulating factor is an indicator of poor prognosis in sepsis. Intensive Care Med.

[28] Spight D, Trapnell B, Zhao B, Berclaz P, Shanley TP (2008). Granulocyte-macrophage-colony-stimulating factor-dependent peritoneal macrophage responses determine survival in experimentally induced peritonitis and sepsis in mice. Shock.

[29] Bo L, Wang F, Zhu J, Li J, Deng X (2011). Granulocyte-colony stimulating factor (G-CSF) and granulocyte-macrophage colony stimulating factor (GM-CSF) for sepsis: a meta-analysis. Crit Care.

[30] Austin OM, Redmond HP, Watson WG, Cunney RJ, Grace PA, Bouchier-Hayes D (1995). The beneficial effects of immunostimulation in posttraumatic sepsis. The Journal of surgical research.

[31] Fujita Y, Matsuoka N, Temmoku J, Furuya-Yashiro M, Asano T, Sato S, Matsumoto H, Watanabe H, Kozuru H, Yatsuhashi H (2020). JAK inhibitors impair GM-CSF-mediated signaling in innate immune cells. BMC Immunol.

[32] Ge Y, Huang M, Yao YM (2020). Biology of interleukin-17 and its pathophysiological significance in sepsis. Front Immunol.

[33] Kim HO, Kim HS, Youn JC, Shin EC, Park S (2011). Serum cytokine profiles in healthy young and elderly population assessed using multiplexed bead-based immunoassays. J Transl Med.

[34] Koga T, Migita K, Kawakami A (2016). Biologic therapy in familial Mediterranean fever. Mod Rheumatol.

