# Supplementary material for: Granulocyte-macrophage colony-stimulating factor and tumor necrosis factor-α in combination is a useful diagnostic biomarker to distinguish familial Mediterranean fever from sepsis
Source: Arthritis Res Ther. 2021 Oct 15;23:260. doi: 10.1186/s13075-021-02644-2 (PMC8518289; doi:10.1186/s13075-021-02644-2)
Supplement: Supplementary file 1 — Additional file 1: Supplementary Table S1 Age adjusted ROC curve in each subset determined by multivariable logistic regression analysis [file 13075_2021_2644_MOESM1_ESM.docx]

**Supplementary Table S1. Age adjusted ROC curve in each subset determined by multivariable logistic regression analysis**

| variables (FMF vs sepsis) | sensitivity | specificity | accuracy | AUC | AIC | cut-off value  (predicted probability) |
| --- | --- | --- | --- | --- | --- | --- |
| GM-CSF + age | 0.85 | 1 | 0.91 | 0.97 | 27.0 | 0.85 |
| TNF-α + age | 0.85 | 0.86 | 0.85 | 0.86 | 46.1 | 0.66 |
| VEGF + age | 0.89 | 0.75 | 0.84 | 0.88 | 42.0 | 0.48 |
| GM-CSF + TNF-α + age | 0.93 | 1 | 0.95 | 0.99 | 19.9 | 0.79 |
| GM-CSF + VEGF + age | 0.85 | 1 | 0.91 | 0.97 | 29.0 | 0.83 |
| GM-CSF + age | 0.85 | 1 | 0.91 | 0.97 | 27.0 | 0.85 |

Bold indicates the minimum number of cytokines among the subsets. AIC = Akaike's information criterion; AUC = area under the curve; GM-CSF = granulocyte macrophage colony stimulating factor; TNF-α = tumor necrosis factor-alpha; VEGF = vascular endothelial growth factor.
